# Multimodal Movement Prediction - Towards an Individual Assistance of Patients

**DOI:** 10.1371/journal.pone.0085060

**Published:** 2014-01-08

**Authors:** Elsa Andrea Kirchner, Marc Tabie, Anett Seeland

**Affiliations:** 1 Robotics Lab, University of Bremen, Bremen, Germany; 2 Robotics Innovation Center (RIC), German Research Center for Artificial Intelligence (DFKI), Bremen, Germany; Katholieke Universiteit Leuven, Belgium

## Abstract

Assistive devices, like exoskeletons or orthoses, often make use of physiological data that allow the detection or prediction of movement onset. Movement onset can be detected at the executing site, the skeletal muscles, as by means of electromyography. Movement intention can be detected by the analysis of brain activity, recorded by, e.g., electroencephalography, or in the behavior of the subject by, e.g., eye movement analysis. These different approaches can be used depending on the kind of neuromuscular disorder, state of therapy or assistive device. In this work we conducted experiments with healthy subjects while performing self-initiated and self-paced arm movements. While other studies showed that multimodal signal analysis can improve the performance of predictions, we show that a sensible combination of electroencephalographic and electromyographic data can potentially improve the adaptability of assistive technical devices with respect to the individual demands of, e.g., early and late stages in rehabilitation therapy. In earlier stages for patients with weak muscle or motor related brain activity it is important to achieve high positive detection rates to support self-initiated movements. To detect most movement intentions from electroencephalographic *or* electromyographic data motivates a patient and can enhance her/his progress in rehabilitation. In a later stage for patients with stronger muscle or brain activity, reliable movement prediction is more important to encourage patients to behave more accurately and to invest more effort in the task. Further, the false detection rate needs to be reduced. We propose that both types of physiological data can be used in an *and* combination, where both signals must be detected to drive a movement. By this approach the behavior of the patient during later therapy can be controlled better and false positive detections, which can be very annoying for patients who are further advanced in rehabilitation, can be avoided.

## Introduction

The application of robotics for neuromotor rehabilitation is a very challenging but also promising approach [1]. Today, there are already robotic systems that are applied for upper and lower limb therapy and positive effects on rehabilitation progress could be shown [2]–[6]. Any therapy is, however, only effective if patients do accept it and have a positive attitude towards it. This is especially true when assistive technology devices (definition see [7]) are applied. To improve acceptance of an assistive technology, a device must not restrict the person who is wearing it and must be comfortable and intuitive to use [8]. To support natural behavior, such a device should not be fixed to a special support mechanism, if the state of the patients allows this. Further, it should have multiple contact points to the patient€s body to avoid pressure points and to allow the reflection of complex force patterns for accurate guidance [9].

Besides theses challenges in structural design, the assistive device should support self-initiated movements for intuitive interaction. This can be achieved by adapting the control of the device with respect to the patient’s intention. Movement intention of the patient can be detected from her/his brain activity, e.g., the electroencephalogram (EEG), as shown in healthy subjects [8], [10]–[13] as well as in stroke patients [14], and by the analysis of gaze direction and fixation [15], or by the analysis of the electromyogram (EMG) [16]. EMG activity is quite often solely used to trigger an orthosis or a prosthesis [17]–[19]. EEG activity can alternatively be used to trigger the support by the device in case that muscle activity is largely diminished as it can be observed after peripheral or spinal lesions of nerves [20].

The integration of EEG-based predictions into the control of an assistive technical device has one great advantage: The earliness of prediction that can be achieved based on EEG analysis allows to close the gap between movement planning and execution for natural behavior and may thus boost rehabilitation since the patient gets the feeling as if she/he and not the assistive device, like an exoskeleton, is controlling the limb [21]. Further, certain event-related activities in the EEG are a reliable indicator that a patient wants to execute a self-initiated, voluntary movement. Especially the readiness potential (RP) is only seen before voluntary movements and not before involuntary movements and can thus be used in clinical practice to differentiate between voluntary and involuntary movements [22].

**Figure 1:**
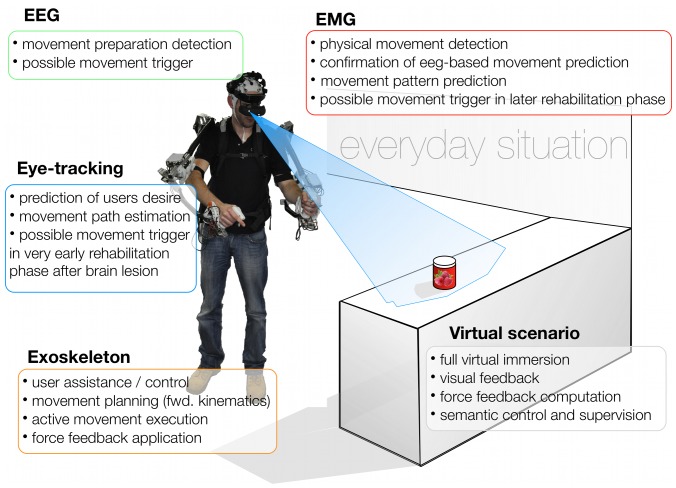
Schemata of a subject assisted by an exoskeleton within a possible rehabilitation scenario. The exoskeleton is controlled online via signals directly recorded from the user and an eye-tracking system. The support is context driven and can be realized in the real world or a virtual scenario.

However, it is not always useful to rely on EEG-based predictions alone. Different kinds of physiological data, but also technical data that is recorded by the assistive technical device [9], [23], can be combined to improve the reliability of EEG-based predictions as well as its fully automated application [24]. A prediction of movement onset that was made based on EEG analysis can for example be confirmed by (i) a simultaneously detected fixation of manipulable objects by the eyes, (ii) the detection of muscle activity or by (iii) measuring pressure against force sensors of the device. Assistive technology devices that are supported by integrated analysis of physiological or technical data to enable the detection of movement intention can support a patient for self-initiated movements. By analyzing the context of behavior even complex interaction, like grasping a certain object [15], can be triggered and executed by the device (Figure 1). What sources of physiological data should be combined depends on the requirements, e.g., the kind of disability and neuromuscular disorder [25] as well as the state and progress of the patient in rehabilitation. Further, the correctness of a prediction can in principle be improved by combining several sources of physiological data or even other information, like preferences of the patient [15], [23], [25]. Moreover, it is important to evaluate what effect a combination has on the control of the device. The usage of certain data may prohibit an early prediction. For example, the electrooculogram (EOG) or eye tracking can be used to improve the detection performance of EEG-based predictions [15]. However, a controlled eye movement that can be detected by eye tracking takes place after the subject’s decision to move. Hence, it does not allow the detection of preconscious movement intention [22], but conscious movement intention which is “communicated” by eye movements. To add this signal can still be a good choice for patients whose EEG does not allow good prediction performance or who show no other movement related activity, like EMG, at all, but it does no longer support the positive effect of a fast, almost preconscious control where the subject gets the impression that the device “knows” what her/his intention is. Which signals are relevant at which state of movement planning and execution has been systematically investigated for the prediction of movement targets. For example, in Nowak et al. [15] it was shown that different measures should be combined for different states, e.g., for movement planning, start of movement and movement execution. When different objects were shown to the subject but movement did not yet start, EEG and EOG were found to be most predictive, while eye tracking and EMG could be used best to predict the choice of the target after the movement started.

In this paper, we do not investigate which target a subject wants to reach [15], [25] but rather whether she/he wants to start a movement at all. In Novak et al. [15] movement onset was triggered by cues given by the experimental setup (i.e., auditory cues). In our experiments, subjects performed self-initiated movements. No cue was given to trigger the movement onset. Only a minimum time of rest between movements was demanded. Hence, with the recorded data we were able to investigate, whether it is possible to predict the onset of voluntary movements. For movement onset prediction we analyzed EEG and EMG activity. For both kinds of physiological data it has been showed that a prediction of movement onset is possible before the physical movement starts [8], [10], [13]. It was not the goal of this paper to show that multimodal analysis improves absolute prediction performance but that a different combination of multimodal data, i.e., EEG and EMG data, can help to better adapt an assistive technical device, like an exoskeleton or orthosis, to different states in rehabilitation. Thus, the goal was to show that the functionality of the whole system can maximally be optimized with respect to two different types of errors that have different relevance in different states of rehabilitation. This can be achieved by two different approaches of combining the two kinds of physiological data. Our results show that a different combination of EEG and EMG analysis can either enhance (i) the reliability of movement detection, i.e., decrease the false positive rate (*FP-rate*) (error type I) or (ii) improve the positive detection rate of self-initiated movement detection, i.e., decrease the false negative rate (*FN-rate*) (error type II). The scope of this paper was not yet to show a working approach to support patients nor an online application but an offline analysis of general feasibility. To assure that our results will not be affected by implications of different neuromuscular disorders, we investigated the feasibility of the above explained approach by conducting experiments with healthy subjects. In summary, we show here that a technical device can potentially be adapted by different combinations of EEG and EMG signals to support a patient more individually. This can be applied to adapt the support given by a technical device with respect to the kind of neuromuscular disorder and to her/his state in therapy.

## Materials and Methods

### Experimental Setup

**Figure 2:**
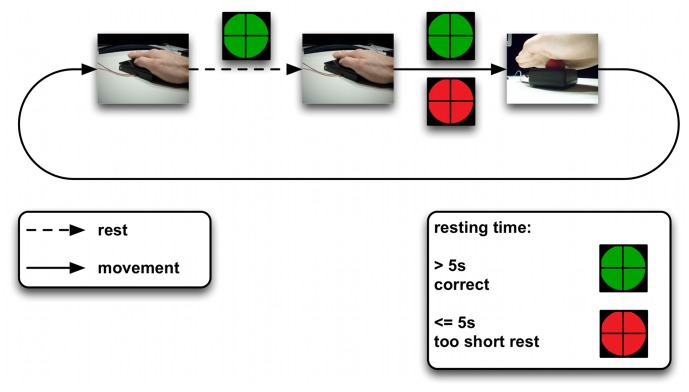
Schemata of the conducted experiments. Subjects were asked to move their right hand from a flat micro switch board to a buzzer, while looking at a green fixation cross presented on a PC-monitor. Between two consecutive movements a minimum resting time of 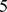 s had to be maintained. Too early movements were reported to the subjects by changing the color of the fixation cross to red for 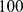 ms. After 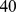 valid movements one complete run was finished.

Eight healthy male subjects (age: 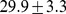 years; right-handed; normal or corrected-to-normal vision) participated in the study. The subjects were seated in a comfortable chair in front of a table. A monitor was used to give feedback to the subjects. The subjects executed self-initiated and self-paced movements of the right arm (Figure 2). Further, two input devices containing micro switches were used to monitor the beginning and end of performed movements. The input devices were placed at a distance of approximately 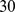 cm from each other. Events that were detected by the devices (pressing/releasing) were marked in the EEG/EMG data. Subjects were asked to move their hand from one input device to the other and back in their own speed, to produce natural movements. During the experiments a green circle with a black fixation cross was shown to the subjects on the monitor. For each subject we recorded 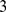 runs. Executed movements were self-initiated and the only constraint for two consecutive movements was a resting time of at least 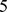 s. Movements carried out too early were reported to the subjects by changing the color of the green circle to red for 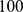 ms. Such wrong movement trials were not used for later data analysis. A run ended after 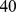 correctly performed movements. The experiment was designed with Presentation [Neurobehavioral Systems, Inc., Albany, USA].

#### Ethics statement

The study has been conducted in accordance with the Declaration of Helsinki and approved with written consent by the ethics committee of the University of Bremen. Subjects have given informed and written consent to participate.

### Data Acquisition

EEGs and EMGs were acquired with 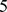 kHz, filtered between 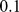 to 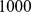 Hz using BrainAmp DC (EEG) and BrainAmp ExG MR (EMG) amplifiers [Brain Products GmbH, Munich, Germany] and saved to a computer. EEGs were recorded with a 128-channel actiCap system (reference at FCz) and EMGs were measured bipolar with Ag/AgCl gel electrodes at four muscles of the right arm: M. brachioradialis, M. bizeps brachii, M. triceps brachii, and M. deltoideus. Events from the two input devices (see Section “sec:expset”) were labeled in the recorded data. A motion capturing system was used to detect the physical movement onset of the subjects right arm. The system consisted of three cameras (ProReflex 1000) [Qualisys AB, Gothenburg, Sweden] and a passive infrared marker mounted on the back of the test persons right hand. Motions of the right hand were recorded with a sampling frequency of 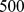 Hz.

### Data Processing

#### Estimation of physical movement onset

In an offline analysis the EEG and EMG data as well as the data from the motion tracking system were synchronized and the time points of the physical movement onsets were extracted from the tracking data. As described in Section “sec:expset”, the beginning of each movement was labeled in the EEG/EMG data by a microswitch. It is obvious that the subject was already in the movement phase when the switch had been released, since a little lift of the hand is required for this. Therefore, the data from the motion tracking system was analyzed in order to find the correct physical movement onset. First, the movement speed of the subject’s hand was calculated from the movement tracking data by computing the euclidean distance of consecutive samples. The unit of this speed is in mm/sample; the sampling period is 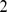 ms. Then, starting from each microswitch label, the movement speed for each movement trial was analyzed backwards. The physical movement onset was set as soon as the movement speed was below a threshold of 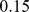 mm/sample, since this is the given accuracy of the tracking system. The determined time points were labeled in the EEG/EMG data, and used as the ground truth for the beginning of a movement in the data analysis.

#### EEG analysis

For EEG data analysis, 64 of the 128 recorded channels (extended 10–20 system) were used. The analysis of the EEG data was optimized to detect event-related potentials (ERPs) in single trial. Movement planning evokes several movement-related cortical potentials [26]. The earliest that can be detected is the Readiness Potential (RP). For unilateral movements, the RP is a slow negative shift bilaterally widespread over the parietal and precentral cortex. The RP begins symmetrically, the late RP for unilateral movements then has a contralateral preponderance [27]. Both, early and late RP, also called Lateralized RP (LRP) [28], [29], are associated with the planning phase of a movement. An early detection of these components allows the prediction of movement onset [10], [14].

For preprocessing, the data was standardized channel-wise (subtraction of mean and division by standard deviation (SD)) and decimated to 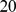 Hz. Next, a FFT band-pass filter with a pass band of 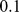 to 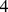 Hz and xDAWN, a spatial filter [30], were applied. The xDAWN spatial filter is especially designed for enhancing the synchronous response of ERPs. It is assumed that the data can be described as the true ERP occurring at predefined events plus some noise. In the algorithm [30], this true ERP matrix is estimated using a least squares approach. The filter matrix that contains a linear combination of the original channels (pseudo-channels) in each column is then calculated by maximizing the signal-to-signal-plus-noise-ratio (given by the generalized Rayleigh coefficient). Since signal and noise are separated in this way, dimensionality reduction is possible, i.e., only a few pseudo-channels are sufficient to explain most of the variance in the data. Here, four pseudo-channels were used. The data was processed window wise on windows with a length of 1000 ms. The last four samples of the windows were used as time domain features and a Gaussian-Feature-Normalization (features have zero mean and variance one) was performed. For later classification, a support vector machine (SVM) [31] was trained.

Training windows were defined for both classes: “movement intention” and “resting state”. For “movement intention” the windows 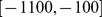 ms and 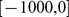 ms before each physical movement onset were used. For “resting state” windows were cut every 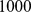 ms, as long as no movement occurred 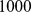 ms before and 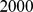 ms after a window.

In the test case, overlapping windows were cut every 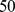 ms in a range from 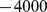 ms to 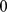 ms before a movement (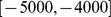 ms, 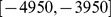 ms, …, 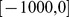 ms). A prediction of a movement was allowed in a range of 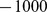 ms to 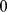 ms before movement onset. As border between classes 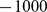 ms with respect to the physical movement onset was chosen, although it is known that the RP [28], [29] that is detected by the performed analysis can be expressed way before 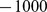 ms or later [26]. We considered a) the signal properties and b) a possible application for our choice of the class border. In the application we aim at enabling the assistive device to start the movement simultaneously with the patient’s conscious will to move. Hence, detection of unconscious movement intention is only useful if the device needs time for reaction. Very early detections of movement intention might lead to a triggering of movement onset by the assistive device before the patient is ready. The obtained SVM scores were transformed to a movement probability with a sigmoid function [32]. A probability greater than 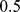 corresponded to movement preparation. For each subject individually a 3-fold cross validation analysis of the data was performed, in which each fold corresponded to one experimental run. During the training phase the complexity parameter of the SVM was optimized using a grid search. The grid contained 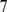 values: 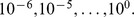


#### EMG analysis

For EMG analysis all four recorded channels and additionally the mean of all channels were used as different possible input sources. The data was preprocessed with a variance filter, defined as
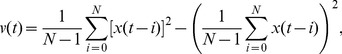
(1)with, 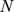 being the length of the window used for filtering and x the raw EMG signal. The variance was chosen for preprocessing, since it incorporates filtering and feature generation abilities. Classification was done using an adaptive threshold, defined as



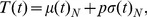
(2)with 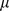 being the mean value, 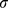 the standard deviation, 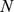 the length of the window for the mean and standard deviation and 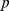 the sensitivity factor of the threshold. The adaptive threshold is used due to its capability of compensating slow drifts in the EMG signals or possible higher noise levels in the signal caused, for example, by resistance changes at the electrode side. Similar to the EEG analysis a cross validation was used for training and testing. During the training phase the parameters for the filter and the threshold were optimized and the best channel was chosen. For the optimization a grid search was used. Further details are given in [16].

For the EMG-based prediction of movement onset a detection of EMG activity was allowed in the range of 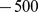 ms to 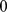 ms before the physical movement onset. Although for EMG data analysis this lower bound (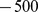 ms) is not supported by the signal characteristic, we found that EMG can be detected quite early in case of a pre-load of muscle activity in preparation of the movement onset. Considering this and after inspection of the data sets with respect to pre-load in muscle activity, 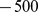 ms was found to be an appropriate border for all subjects to cover most of the relevant EMG onsets. Such early EMG activity must be detected in a potential online case to control a device. An early detection of EMG activity would further support an early prediction of movement onset in the AND condition, which was intended to give the subjects the feeling of fast response of the device in case that the approach would be applied online.

### Definition of Conditions

We investigated four different conditions:

**A:** EEG-based prediction. In this condition prediction of movement onset is based only on EEG analysis.

**B:** EMG-based prediction. In this condition prediction of movement onset is based only on EMG analysis.

**C:** “OR” combination of A and B. Here A and B are combined in a way that a movement onset counts as predicted, if either EEG *or* EMG-based analysis or both predicted a movement.

**D:** “AND” combination of A and B. Here A and B are combined in a way that a movement onset counts as predicted, if both EEG *and* EMG-based analysis predicted the movement.

For all conditions the *TP-* and *FP-rates* as well as the balanced accuracy (BA) and the mean prediction times were calculated.

### Performance Metrics

As performance metrics we used the true positive and false positive rate, defined as 
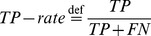
(3)and
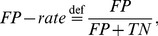
(4)where TP is the number of correctly classified “movement intention” windows, FN is the number of wrongly classified “movement intention” windows, TN is the number of correctly classified “resting state” windows and FP is the number of wrongly classified “resting state” windows, respectively. Note that for calculating the 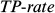 one correctly classified window based on EEG analysis in the range of 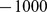 ms to 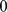 ms and for EMG in the range of 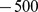 ms to 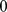 ms was sufficient. For the 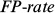 each window from the “resting state” that wrongly predicted a movement was counted as FP.

Note that from a statistical point of view it is more intuitive to compare two error types, rather than an error type (FP-rate) with a success type (TP-rate). However, due to the relation between the *TP-* and *FN-rate* (error type II is defined as 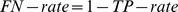) the statistical results obtained by using the *TP-* and *FP-rate* is equivalent to that provided by using the *FN-* and *FP-rate*. Thus, to compare the two different signal types and their combinations, the error rates obtained were analyzed by repeated measures ANOVA with error type (*FP-rate*: error type I/*FN-rate*: error type II) and signal type and their combinations (EEG/EMG/“AND”/“OR”) as within-subjects factors. Where necessary, the Greenhouse–Geisser correction was applied and the corrected 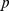-value is reported. For multiple comparisons, the Bonferroni correction was applied.

As a common metric to evaluate the performances obtained from the two types of signals (EEG/EMG) and their combinations (AND/OR) together, we used the BA, defined as:
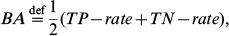
(5)where 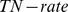 is the true negative rate equal to 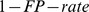. The BA is calculated as balanced classification rate (i.e., the BA considers the accuracy of the positive class and accuracy of the negative class independently) and thus the BA is insensitive to unbalanced class ratios. Such unbalanced ratios between the positive and negative class have to be considered, since in this study 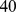 “movement intention” and 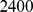 “resting state”examples occurred per run. It is important to show that the approach of combining both EEG and EMG signals for adapting an assistive technical device with respect to the requirements of therapy does not influence absolute prediction performance too negatively. However, it should be noted that the two methods (EEG- and EMG-based movement prediction) behave differently concerning the ratio between *FP-rate* and *FN-rate*. More specific, we observed similar levels of both error rates for the EEG, while these levels were very different for the EMG signal (see Table 1). Hence, it is not straightforward to compare the two different signal types (EEG/EMG) and their combinations (AND/OR) with a single metric that does not take into account these observed differences. To still enable a direct comparison we provide the BA values. Other metrics can be calculated based on the given performance rates.

**Table 1:** Classification results for all 4 conditions.

Condition	A (EEG)	B (EMG)	C (“OR”)	D (“AND”)
TP-rate	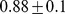	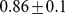	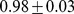	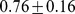
FP-rate	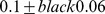	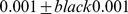	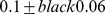	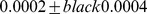
TN-rate	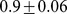	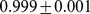	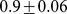	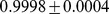
FN-rate	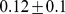	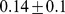	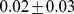	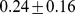
balanced accuracy	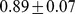	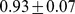	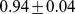	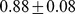
prediction time (ms)	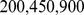	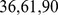	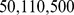	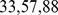

Results for different classification conditions (from left to right: only EEG, only EMG, combination of both with “OR” and with “AND”): The mean classification results with standard deviation are shown in TP-, FP-, TN-, FN-rate and balanced accuracy. The prediction time is given in 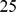 %–, 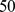 %– and 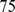 %–quantiles, respectively.

### Prediction Time

The prediction time is defined as the earliest point in time (under the above defined conditions, i.e., interval boundaries) where a physical movement start could be predicted. Since the distributions of prediction times differ a lot for EEG and EMG-based classification and especially the combination (condition C, see above) is not Gaussian distributed, we report median and quartiles here. For EMG-based predictions of movement onset (condition B, see above) the point in time was marked at which the adaptive threshold is exceeded. For EEG-based detection of movement intention (condition A, see above) the earliest time window (furthest away from movement onset) that was classified to belong to the class of movement intention was marked.

## Results

### Prediction Time

**Figure 3:**
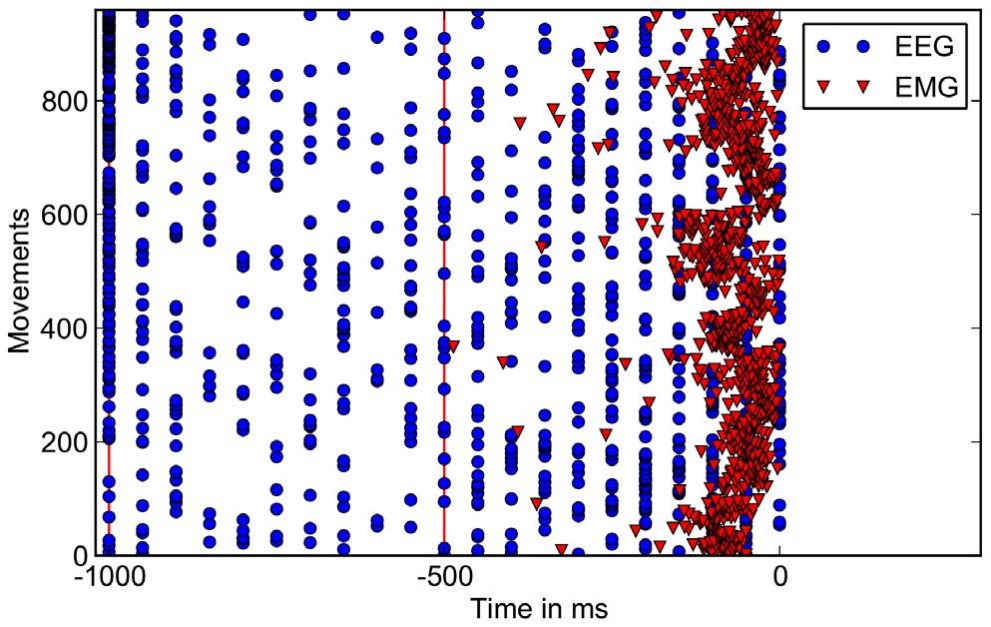
Distribution of prediction times for EEG-based (blue) and EMG-based (red) movement prediction. Time point zero corresponds to the physical movement onset, the red line at time 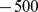 ms indicates the range up to where predictions based on EMG were allowed, for EEG predictions up to 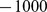 ms before physical movement onset were allowed, again marked with a red line.

**Figure 4:**
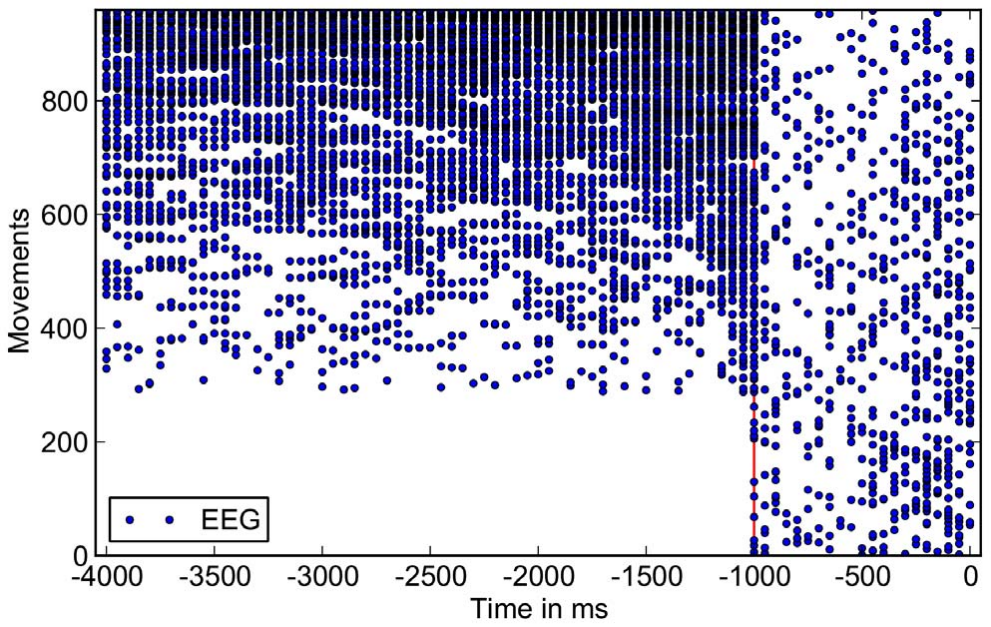
Distribution of prediction times for EEG-based movement prediction including the non movement range. Time point zero corresponds to the physical movement onset, the red line at time 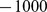 ms indicates the range up to where predictions based on EEG were allowed, the range from 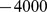 ms to 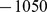 ms corresponds to the no movement class, hence all predicted windows in that range count as FPs.

In Figure 3 the distribution of prediction times for EEG and EMG-based predictions of movement onset is shown (see also Table 1). For visualization purposes we provide a video as supporting information that is visualizing the performance of the different movement prediction conditions (see Video S1 as supporting information). For EEG-based detection of movement intention the median of the prediction time was 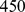 ms (with lower 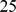
%-quartile 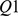: 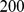 ms and upper 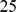
%-quartile 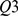: 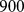 ms), for EMG-based predictions of movement onset the median of the prediction time was 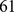 ms (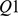: 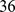 ms, 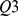: 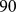 ms). Note that for EEG-based predictions the prediction times are clustered in lines with a spacing of 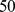 ms due to the windowing procedure explained in Section “sec:dataproc”. In Figure 4 all windows classified as “movement intention” are plotted to better visualize the distribution of false and true positive classification. The plot shows that for EEG - predictions, instances (windows) recorded before 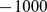 ms were sometimes also classified as movement intention. In the plot, all individual movements (independent of subject and run) are ordered in the way that the ones with the highest *FP-rate* are displayed at the top of the figure. It is obvious that for single movements for which EEG-based movement prediction was too early, the general performance in separating classes (“movement intention” and “resting state”) was weak. In the lower part of Figure 4, 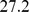 % of the whole 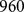 movements that were analyzed in this study contain no FPs (no positive predictions before 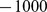 ms with respect to the physical movement onset). For the combination of both signals in an “OR” fashion (condition C) the median of the prediction time was 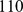 ms (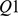: 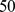 ms, 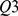: 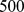 ms) and for the “AND” combination (condition D) the median of the prediction time was 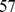 ms (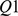: 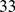 ms, 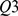: 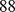 ms).

### Prediction Performance

**Figure 5:**
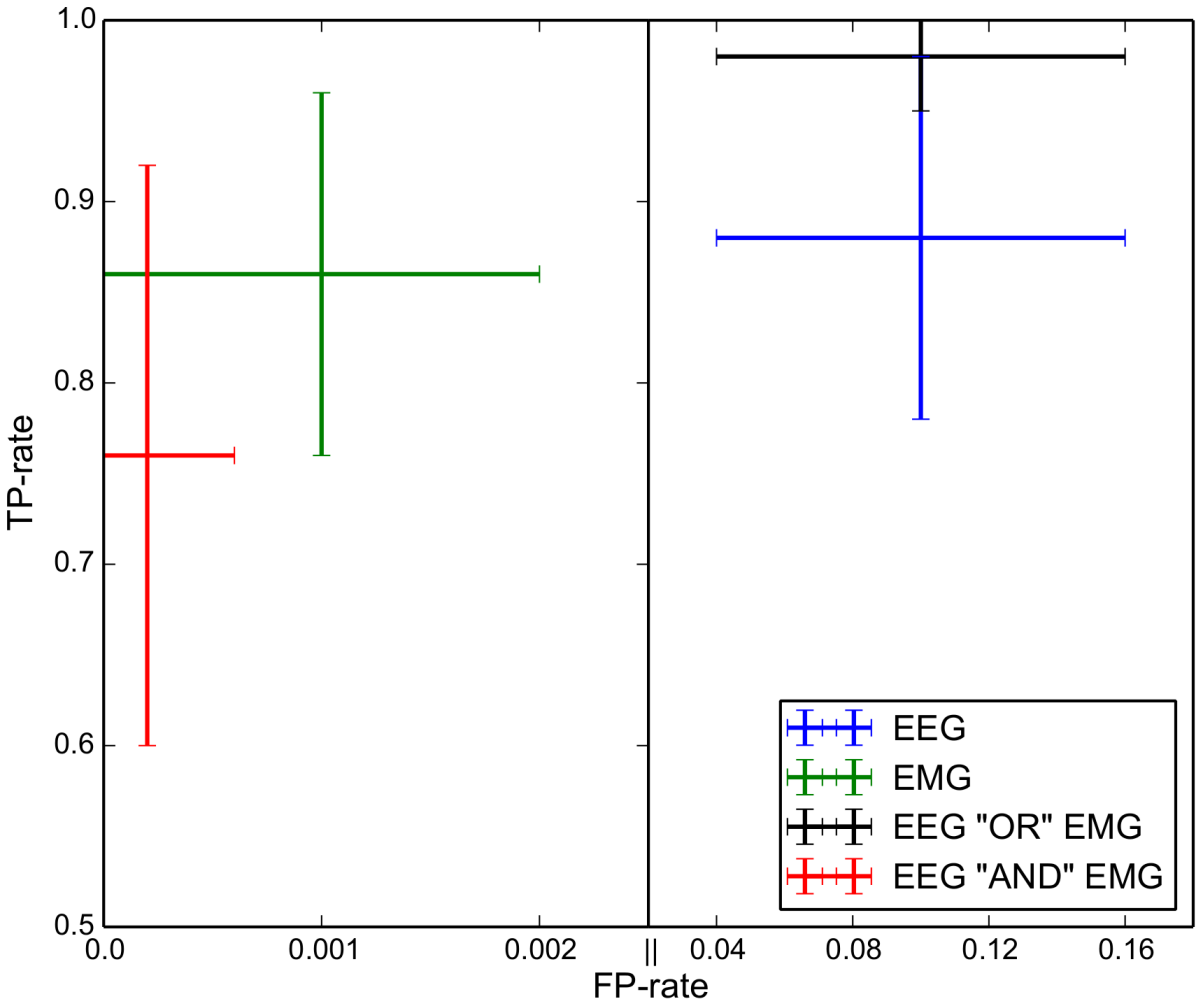
Prediction results in TP- and FP-rate for Condition A (EEG) (blue), Condition B (EMG) (green), Condition C (EEG “OR” EMG) (black) and Condition D (EEG “AND” EMG) (red). The mean TP-rate and FP-rate for all subjects is shown; the bars indicate the standard deviation. Note for the x-axis two scales are used, since the FP-rates for Condition B (EMG) and Condition D (EEG “AND” EMG) are very small compared to the other two conditions. The two vertical dashes within the x-axis label highlight the scale change.

**Figure 6:**
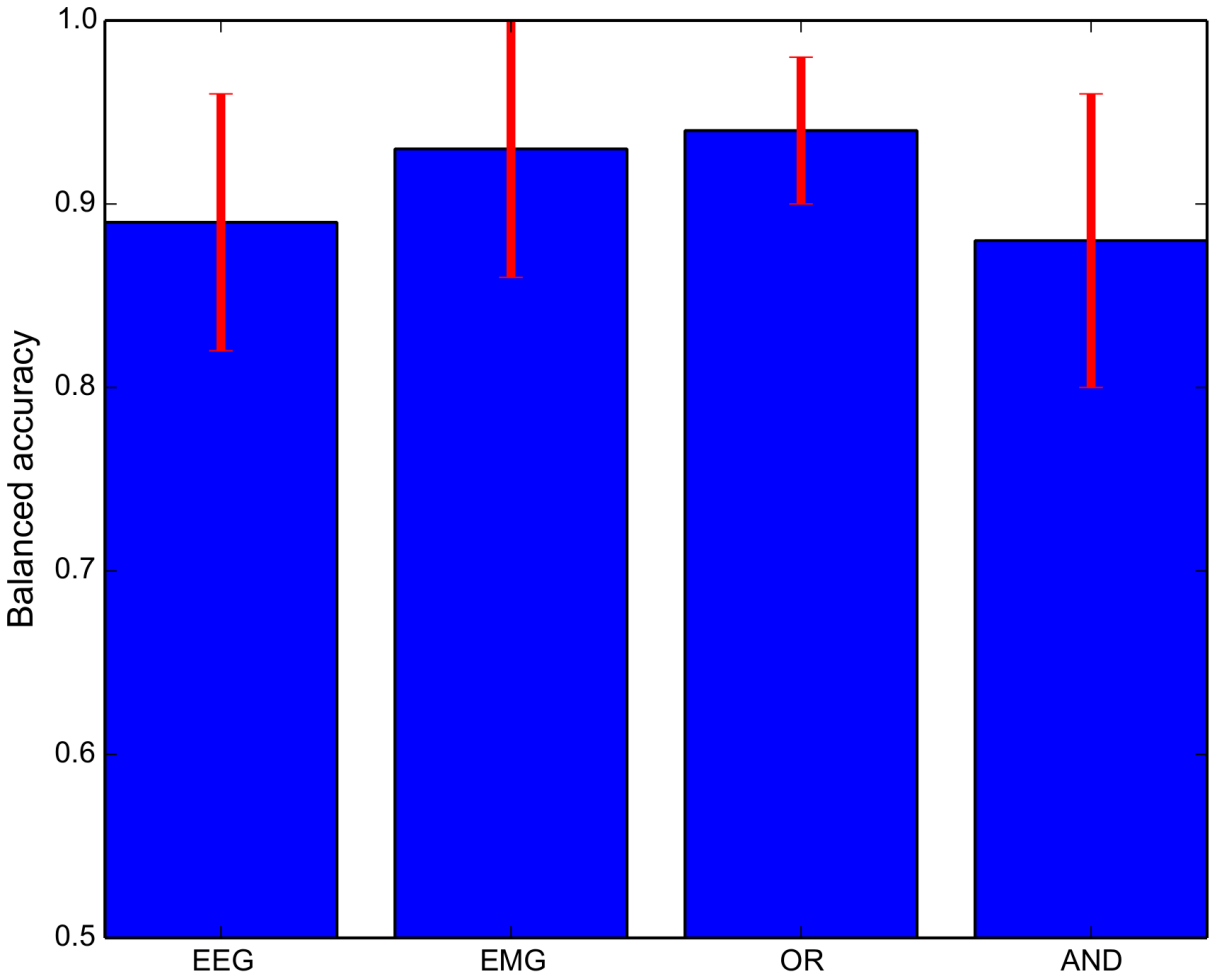
Prediction results in balanced accuracy for Condition A (EEG), Condition B (EMG), Condition C (EEG “OR” EMG) and Condition D (EEG “AND” EMG). The mean balanced accuracy for all subjects is shown; the bars indicate the standard deviation.

The classification results are summarized in Table 1 and visualized in Figure 5 (*TP−/TN-* and *FP−/FN-rates*) and Figure 6 (balanced accuracy). Statistical analysis revealed that the signal types and their combinations (EEG/EMG/“AND”/“OR”) affect the error rate for both types of error (*FP-rate*: error type I/*FN-rate*: error type II) [interaction between error type and type of signal combinations: 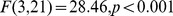]. For the error type I, the “AND” combination is the best combination of signals, i.e., reduces error of type I most, with significant differences to all other types of signal and their combinations [AND vs. EMG: 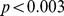, AND vs. EEG: 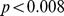, AND vs. OR: 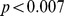]. The type of signal “EMG” is better than the type of signal “EEG” [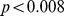] and the “OR” combination [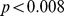]. The type of signal “EEG” is better than the “OR” combination [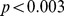]. For the error type II the “OR” combination is the best combination of signals, i.e., reduces error of type II most, with significant differences to all other types of signal and their combinations [OR vs. EMG: 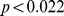, OR vs. EEG: 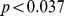, OR vs. AND: 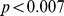]. The type of signal “EMG” is better than the “AND” combination [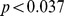], but not better than the type of signal “EEG” [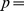 n.s.]. The type of signal “EEG” is better than the “AND” combination [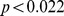].

## Discussion and Conclusion

Results show that both, EEG and EMG, signals can be used to reliably predict movements before a physical movement onset. Thus we are able to show in healthy subjects that both signals can potentially be used to control a device with high performance. In case that a fast control algorithm for the assistive device is used [12] the evaluated prediction time would for both conditions A and B allow to support movements in a way that subjects would possibly not notice a delay between their intention and the execution by the device. Since EEG-based predictions can be made much earlier than EMG-based predictions, EEG might be more suitable to give the user the feeling that a device is delivering support on time and without delay. However, whether there is indeed a subjective difference between both methods has to be investigated further.

On the other hand, we showed that EEG analysis can lead to more false positives than EMG analysis does (Figure 5). There are different explanations for this. First, a most important reason for higher 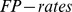 in EEG-based predictions is that movement planning (movement intention) might be detected, which may not result in movement execution [28], [29]. Second, for some subjects EEG-based predictions did not work that well (as supporting information individual results for the prediction time based on EEG analysis are given in Fig. S1) and thus some subjects might have worsened the overall error rates. In this study we did not evaluate this subject-specific effect since we focused on a general evaluation of the potential of combining different methods for adapting an assistive technical device with respect to the state of therapy.

Our results show the capability of multimodal control of assistive devices using a combination of different physiological data on the example of EEG and EMG. It was already shown by other studies that the combination of different measures can improve the performance of detections about subjects intentions, e.g., in the case of movement target prediction [15], [25]. To detect the intention of patients is highly relevant to support them appropriately. A device can best support a movement if, for example, the target of the movement is known. Here we investigated which methods and combination of methods can best be used to predict *when* a patient wants to execute such a movement with respect to different therapy states. To detect movement intention is highly relevant to support self-initiated, voluntary movements by assistive technology devices like active exoskeletons. Our results show, that all classification modalities have a high performance in a range of 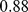 to 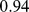 BA. The best result could be achieved using the “OR” combination and EMG as a single modality. Slightly worse results were obtained from EEG-based classification and the “AND” combination. Regarding the absolute performance measures it is hard to decide which signal shall be used to detect movement intention. The most intuitive idea would be to use the “OR” combination because of the highest performance. However, the EEG-based classification has only a slightly worse accuracy, but movements can be predicted 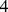 times earlier. Our results show that the signals or combination of signals always need to be chosen according to the application and goals in rehabilitation, as investigated in this work, and are not always solely based on the absolute prediction performance.

During the rehabilitation process the importance of avoiding false movement onset predictions (*FP-rate*; error type I) and thus inappropriate triggering of movements can differ. If the goal of the therapy is to start rehabilitation of patients, who likely produce no strong signals, it is more relevant to detect most movement intentions, hence to reduce the occurrence of type II errors (*FN-rate*). Thus, a combination of signals in an “OR” fashion could be the best choice, since it results in a high 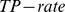 (close to 1 in healthy subjects) and reduces the *FN-rate*, i.e., type II error. By an “AND” combination of both signals on the other hand the 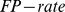, i.e., error type I, can be strongly reduced resulting in a very reliable detection performance. This is desirable as soon as rehabilitation progresses and more precise behavior together with better performance can be expected from the patient. Since the 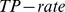 is also reduced (error type II is enhanced), i.e., the patients effort will less likely result in true positive behavior, she/he must try harder to trigger the movement and as a result the engagement of the patient is enforced. Note that it has been shown that the expression of the RP is highly dependent on the motivation of a subject and on how much effort she/he invests [22]. Furthermore, an “AND” combination can help to distinguish voluntary from involuntary movements since only in case of the detection of a RP the assistive device is triggered. Thus involuntary movements will not be supported by the device or could even be diminished by appropriate control mechanisms. Moreover, the “AND” combination reduces the variance in prediction times observed for EEG-based movement prediction, since the variance in EMG-based prediction times was very small.

Results presented here were achieved in an offline combination and comparison of both physiological signals. In future work, we will combine the detection of both kinds of data automatically, to make use of the described advantages in online experiments. Moreover, we want to investigate other ways of combining the two prediction methods, e.g., by applying a classifier that makes use of both (or more) input signals. This can be highly relevant for applying movement prediction from patient’s data to obtain more stable predictions and to cope with the possibly different quality of the measurable signals. Besides this, different methods of analyzing and combing different measures and adding technical measures from the assistive device itself should improve the overall performance as, for example, shown for the prediction of movement trajectories in Corbett et al. [25]. Furthermore, depending on the type of neuromuscular disorder and state of therapy, both, EEG or EMG-based movement prediction, might no longer result in good performance. For example, for patients who suffer from spasm, EMG might no longer be a reliable source for movement onset detection. Here the combination of multimodal data should be even more relevant. This and the level of onset detection performance which would be acceptable for rehabilitation remains to be proven or investigated.

For application in rehabilitation, assistive technical devices must be very easy to use. Most important is the handling, which has to be comfortable for the personnel as well as the patient. It is, for example, very helpful to reduce the amount of electrodes used for recording the physiological signals. For example, in [33] we systematically investigated how many EEG electrodes are sufficient to detect the P300 [34] ERP in single trial. We found that the reduction to even 8 electrodes would not dramatically reduce the prediction performance. For the detection of movement related ERP potentials, an even lower amount of electrodes might be sufficient since the RP and LRP are very local activities that can be recorded at electrodes positions C1/C3 and C2/C4 for hand and arm movements. Further, dry electrodes, which can easily be applied for EMG data acquisition [35], can also be used for EEG data acquisition. Although with dry electrodes it is quite challenging to conduct high quality EEG recordings, some approaches are very promising to reduce the effort of EEG acquisition. For example, a low number of as much as 6 dry electrodes could be shown to be sufficient for the prediction of movement intention [36]. Note that the referenced study was based on signals in the frequency domain and not in the time domain as it was the case here. Hence, results cannot simply be transferred to our study. Furthermore, for patients it might not be straightforward to decide on best electrodes to choose, due to bigger differences between patients, e.g., resulting from brain injury that leads to massive changes in the brain activity. Hence, sophisticated methods that can be applied to automatically find best electrode combinations must be developed which are currently investigated by our group (see for example [37]). We further believe that, if a system, that is supported by EMG data, is applied successfully to most patients, the step is smaller to add EEG in case EMG-based predictions are not sufficient for some patients. By a stepwise integration of different kinds of data, the barrier can be reduced to also integrate measures like EEG for multimodal signal analysis and support. However, some work still remains to be done to increase the general acceptance of assistive technology devices like active exoskeletons for rehabilitation although there are already some positive examples of their application as discussed above. To take the next logical step, we started cooperations with clinical partners to evaluate our approach on patients.

In summary, results presented here support the hypothesis that multimodal analysis of physiological data has the potential to support patients by assistive technology devices more individually to their kind of disease and state of rehabilitation with respect to movement onset detection. We expect that for patients this effect will be even more dominant but this has to be evaluated further, especially with respect to acceptable prediction performances and applicability.

## Supporting Information

Figure S1 **Individual prediction performance.** Prediction of movement onset based on EEG (denoted with blue squares) signals performed differently well for individual subjects. A: Good performance for subject 4 in run 2. B: Bad performance, i.e., many false positive detections, for subject 6 in run 3.
(TIFF)

Video S1 **Simulation of the triggering of movements based on the analysis of different types of signals (EMG and EEG) and their combinations.** The video shows both arms of the subject filmed from the top of the room. Three frames around the video with colors green (condition A), red (condition B) and yellow (condition D) indicate the detection of a movement intention of one particular condition. In addition, three animations are placed below the video, showing a puppet doing a movement similar to the ones performed by the subject. These animations are again coupled to the three above mentioned prediction conditions and thus triggered by corresponding movement predictions. Condition C is implicitly contained in the video, due to the fact that any prediction made either by EEG or EMG is displayed.
(MP4)
