# Supplementary figures and images for: Multimodal Movement Prediction - Towards an Individual Assistance of Patients
Source: PLoS One. 2014 Jan 8;9(1):e85060. doi: 10.1371/journal.pone.0085060 (PMC3885685; doi:10.1371/journal.pone.0085060)

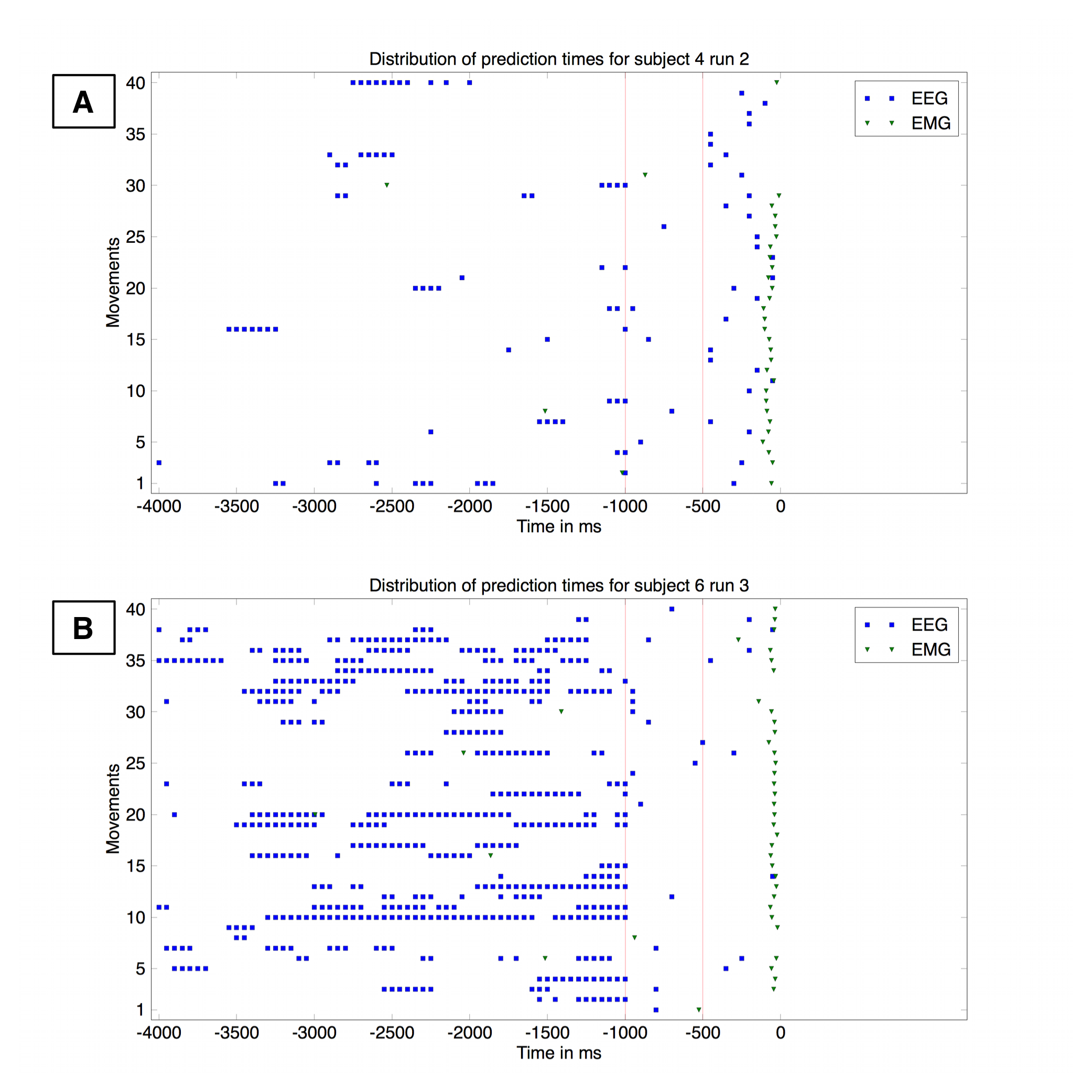

Supplement: Figure S1 — Individual prediction performance. Prediction of movement onset based on EEG (denoted with blue squares) signals performed differently well for individual subjects. A: Good performance for subject 4 in run 2. B: Bad performance, i.e., many false positive detections, for subject 6 in run 3. (TIFF) [file pone.0085060.s001.tiff]
